# Treatment of the humeral shaft fractures - minimally invasive osteosynthesis with bridge plate versus conservative treatment with functional brace: study protocol for a randomised controlled trial

**DOI:** 10.1186/1745-6215-14-246

**Published:** 2013-08-07

**Authors:** Fabio T Matsunaga, Marcel J S Tamaoki, Marcelo H Matsumoto, João B G dos Santos, Flavio Faloppa, João C Belloti

**Affiliations:** 1 Department of Orthopedics and Traumatology, Universidade Federal de São Paulo - Escola Paulista de Medicina (Unifesp-EPM), São Paulo, Brazil

**Keywords:** Humeral fractures, Immobilisation, Fracture fixation, Internal, Orthopaedic fixation devices

## Abstract

**Background:**

Humeral shaft fractures account for 1 to 3% of all fractures in adults and for 20% of all humeral fractures. Non-operative treatment is still the standard treatment of isolated humeral shaft fractures, although this method can present unsatisfactory results. Surgical treatment is reserved for specific conditions. Modern concepts of internal fixation of long bone shaft fractures advocate relative stabilisation techniques with no harm to fracture zone. Recently described, minimally invasive bridge plate osteosynthesis has been shown to be a secure technique with good results for treating humeral shaft fractures. There is no good quality evidence advocating which method is more effective. This randomised controlled trial will be performed to investigate the effectiveness of surgical treatment of humeral shaft fractures with bridge plating in comparison with conservative treatment with functional brace.

**Methods/Design:**

This randomised clinical trial aims to include 110 patients with humeral shaft fractures who will be allocated after randomisation to one of the two groups: bridge plate or functional brace. Surgical treatment will be performed according to technique described by Livani and Belangero using a narrow DCP plate. Non-operative management will consist of a functional brace for 6 weeks or until fracture consolidation. All patients will be included in the same rehabilitation program and will be followed up for 1 year after intervention. The primary outcome will be the DASH score after 6 months of intervention. As secondary outcomes, we will assess SF-36 questionnaire, treatment complications, Constant score, pain (Visual Analogue Scale) and radiographs.

**Discussion:**

According to current evidence shown in a recent systematic review, this study is one of the first randomised controlled trials designed to compare two methods to treat humeral shaft fractures (functional brace and bridge plate surgery).

**Trial registration:**

Current Controlled Trials: ISRCTN24835397

## Background

Humeral shaft fractures account for 1 to 3% of all fractures in adults [1,2] and for 20% of all humeral fractures [3]. These fractures have an annual incidence from 13 to 14.5 per 100,000 people [4,5]. Non-operative treatment is still the standard treatment for isolated humeral shaft fractures [6,7], although this method can present unsatisfactory results, such as, nonunion and shoulder impairment [8,9]. Fourteen percent of patients treated with this method have restricted range of motion and 12.6% have consolidation, with more than 10° of displacement [10].

Surgical treatment is recommended for patients with neurovascular injuries, medullar or brachial plexus injuries, and open fractures, for patients with multiple injuries, and for floating elbow and unsatisfactory reductions [11-13]. Humeral shaft fractures can also be treated surgically for the following indications: *Arbeitsgemeinschaft für Osteosynthesefragen* (AO)-Orthopaedic Trauma Association (OTA) type A fractures, proximal third oblique fractures and distal third shaft fractures [14-16]. Surgical options for treatment of humeral shaft fractures include open reduction and internal fixation with a compression plate, intramedullary nail osteosysthesis and minimally invasive bridge plate fixation. Open reduction and rigid internal fixation with absolute stability using dynamic compression plates [17-19] is today’s standard and is the more common surgical option for treatment of these fractures.

Modern concepts of internal fixation of shaft fractures of the long bones advocate relative stabilisation techniques with no harm to the fracture zone. These have largely been used for fractures of the leg and thigh for which they have become the gold standard treatment. In humeral shaft fractures, these concepts are also being applied with the use of the intramedullary nail [20-22]. In a systematic review, when compared to compression plate osteosynthesis, the use of the intramedullary nail presented a higher risk of shoulder impingement, shoulder pain, and restriction of movements [23]. Recently described by Livani and Belangero [24], minimally invasive bridge plate osteosynthesis with anterior access has been shown to be a secure technique with good results for the majority of humeral shaft fractures [25-27].

Good quality evidence, including trials comparing surgical and nonsurgical interventions for treating these fractures, is lacking [28]. This study will, therefore, be performed to investigate the effectiveness of surgical treatment of humeral shaft fractures with bridge plating in comparison with conservative treatment with a functional brace, considering patients’ superior limb function, their quality of life and treatment complications.

## Methods

This randomised controlled trial will follow the Consolidated Standards of Reporting Trials (CONSORT) Statement [29]; it will be performed in the Hand and Upper Limb Surgery Institute of the Orthopaedics and Traumatology Department of Universidade Federal de Sao Paulo and was approved by the ethical committee (CEP UNIFESP 1595/09). The project is registered in the Current Controlled Trials database (ISRCTN 24835397 http://www.controlled-trials.com/ISRCTN24835397), and was cited by a Cochrane Systematic Review [28]. This study has its funding approved under the process number 2011/21611-2 by a government-based noncommercial agency: Fundação de Amparo à Pesquisa do Estado de São Paulo (FAPESP).

### Inclusion criteria

All patients eighteen years of age or older, with completely deviated humeral shaft fractures (between 4 cm distal to the surgical neck of the humerus and 4 cm proximal to the superior border of the olecranon fossa), who agree to participate and give written informed consent, will be included in the study.

### Exclusion criteria

Patients with pathological or open fracture, previous disease in the limb that could influence the results, an immature skeleton, those whose fracture occurred more than 21 days previously or those with neurovascular-associated injury will be excluded. If patients do not wish to participate or are unable to understand or sign the informed consent form (due to conditions such as cognitive impairment, or mental illness), if poor compliance is expected, or if there any conditions that contraindicate any of the methods for randomized, will also be considered exclusion criteria. Patients who have high risk of anaesthesiology-associated problems will also be excluded.

### Sample size

The sample size was calculated for a significance level of 0.05, statistical power of 90% and SD of 15% in Disability of the Arm, Shoulder and Hand (DASH) scores and an absolute difference between the groups of 10 points in the DASH scores. It was calculated that 50 patients were needed in each group [30,31]. Allowing for a 10% loss to follow up at 24 weeks, we aim to recruit a total of 110 patients.

### Randomisation and allocation

The randomisation sequence will be generated by computer software (http://www.randomizer.org), creating a list from 1 to 110, each number being related to one of the two proposed methods of treatment. We will perform simple (unrestricted) randomisation, making the intervention assignment unpredictable, including the last 10 participants. According to this list, inside each of the 110 opaque sealed envelopes numbered from 1 to 110, will be a piece of paper containing the words ‘functional brace’ or ‘bridge plate.

**Figure 1:**
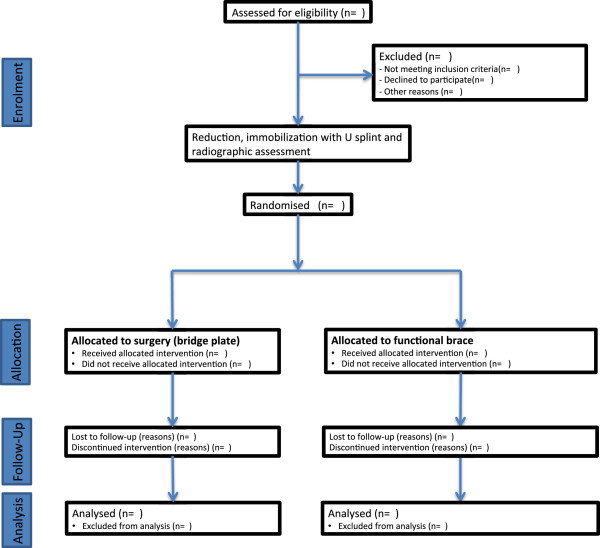
**Flow of participants.** Diagram shows the planned flow of participants through each stage of the study.

Participant allocation will be performed after explaining the protocol and describing both of the procedures to be randomised, and after participants have agreed to take part and signed the informed consent form (Additional file 1 and Additional file 2). They will also be clinically evaluated to determine whether they are suitable candidates for surgery. After this, an independent person will open the envelope before proceeding to the intervention (Figure 1).

### Intervention methods

#### Nonsurgical treatment (functional brace)

**Figure 2:**
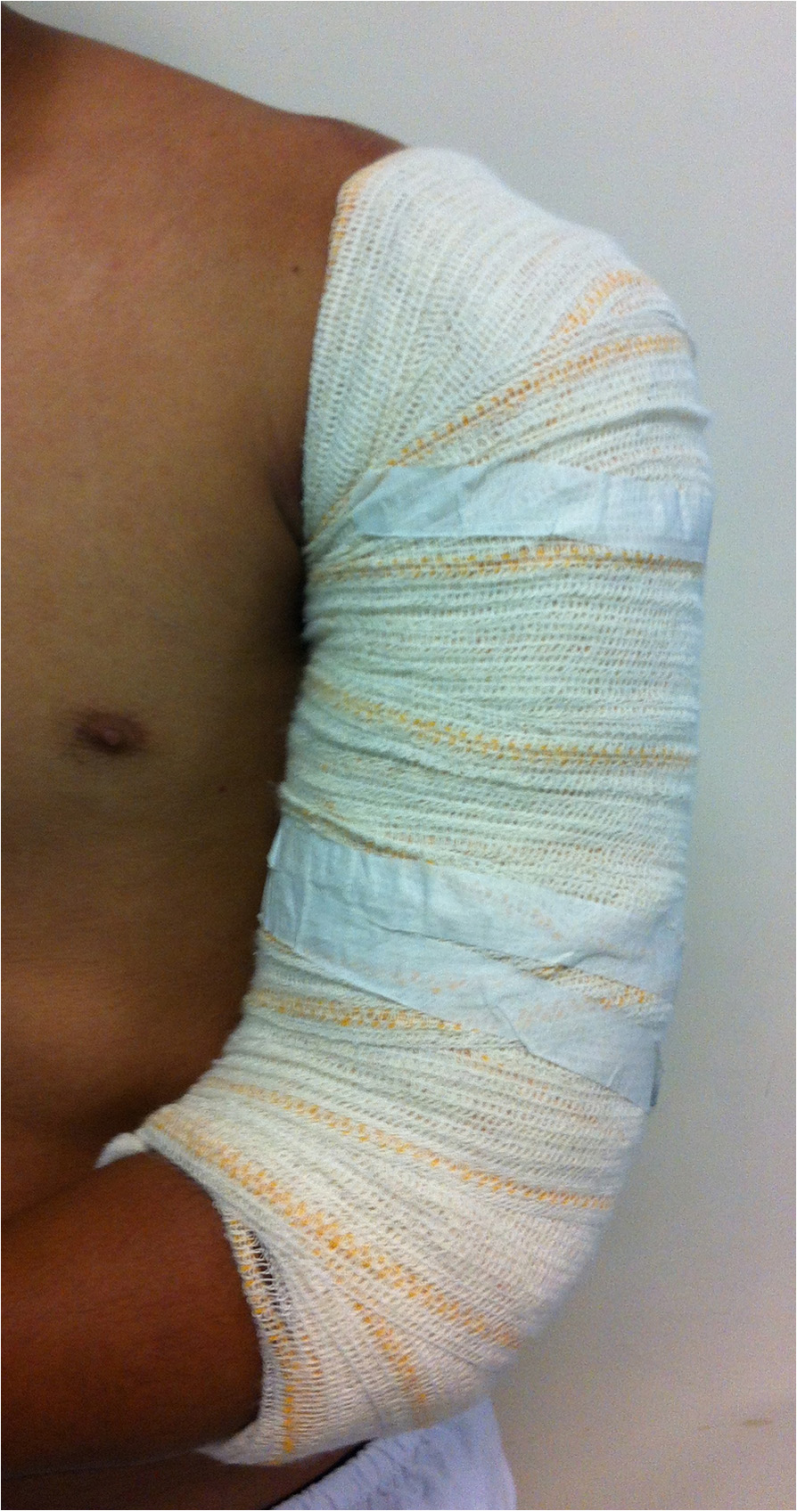
Initial immobilisation with the splint.

**Figure 3:**
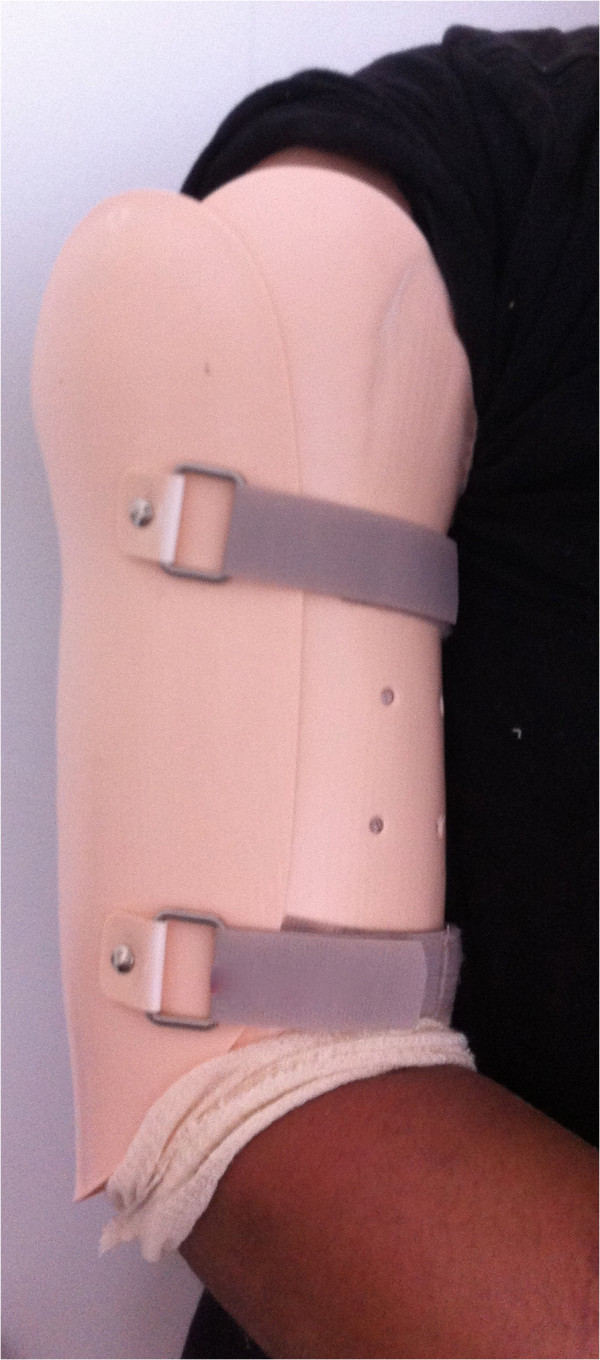
Functional brace.

Patients randomised to nonsurgical treatment will undergo closed reduction and initial immobilisation with a coaptation U-splint [32] (Figure 2) from the axilla to the elbow, ending in the deltoid. After 14 days, the immobilisation will be replaced by a functional brace [6] (Figure 3) allowing the patients to move their shoulder and elbow freely to exercises and rehabilitation. This brace will be kept until fracture consolidation, determined on radiography by two previously-assigned assessors. Any disagreements will be resolved by discussion with a third assessor.

#### Surgical treatment (bridge plate osteosynthesis)

**Figure 4:**
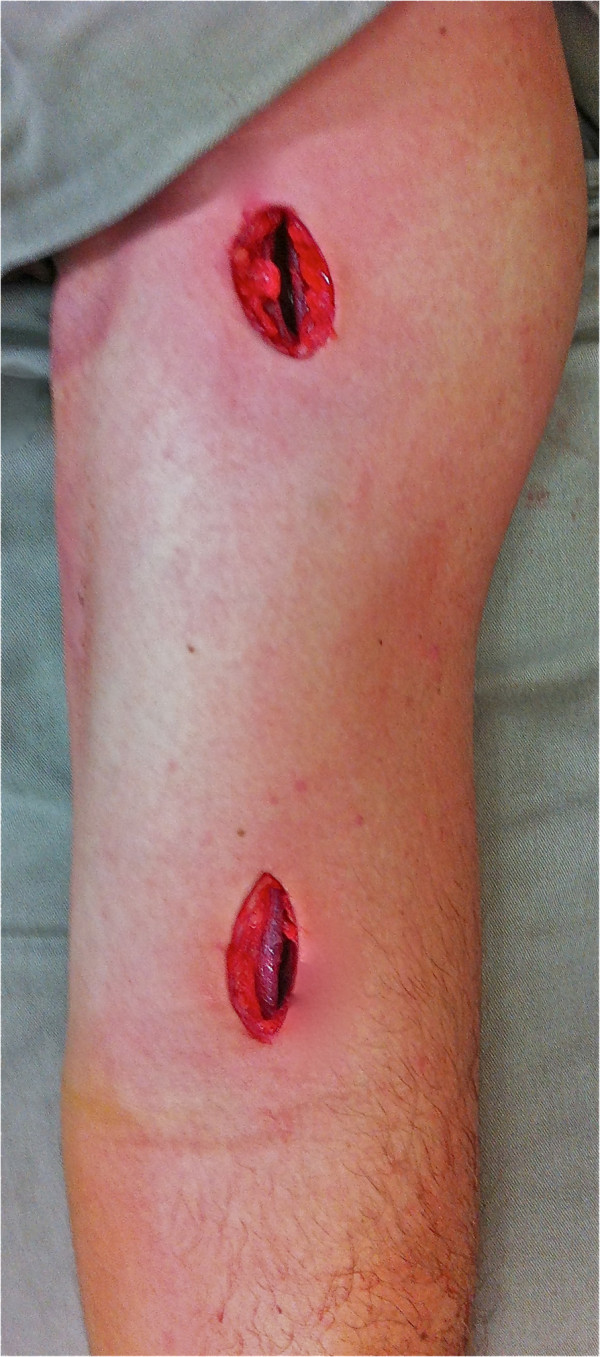
Surgical incisions for minimally invasive bridge plate osteosynthesis.

**Figure 5:**
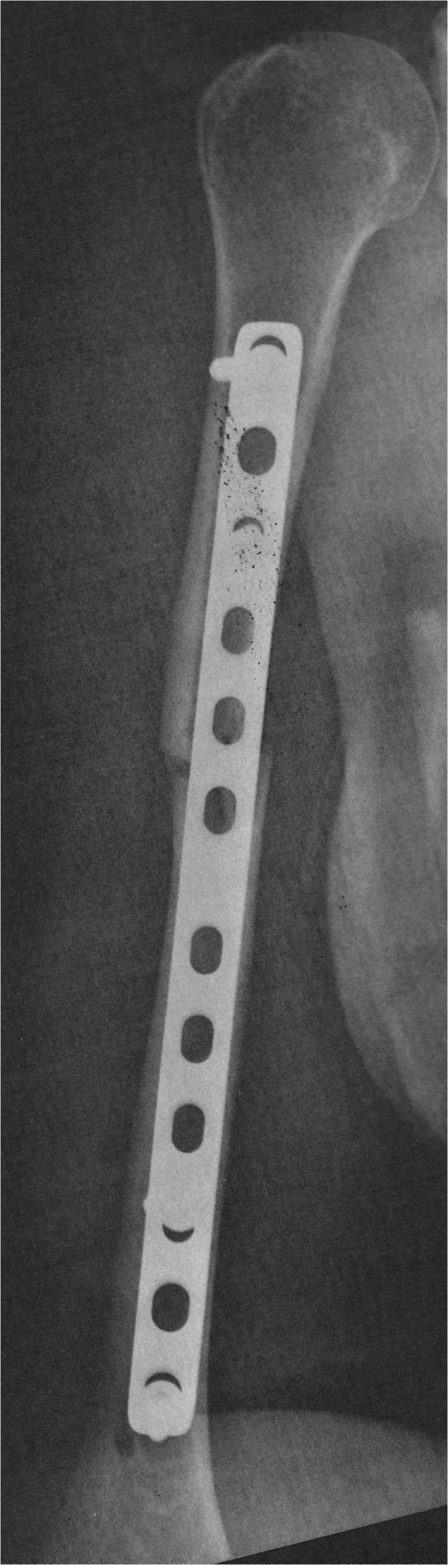
Postoperative radiography.

Patients randomised to surgical treatment will undergo preoperative evaluation of age, clinical condition (acute infection) and co-morbidities. The intervention will take place in the surgical centre of the institution, where four previously-specified surgeons, who are experienced with the surgical technique described by Livani and Belangero [24], will perform the surgical procedures. After the anaesthetic procedure, the patient will be kept in the horizontal dorsal decubitus position and two incisions will be made. The 3 to 5 cm proximal incision will access the proximal fragment between the biceps *brachii* muscle medially and the deltoid muscle laterally. The 3 to 5 cm distal incision will expose the anterior humeral cortex of the distal fragment, after dissection of the lateral cutaneous nerve of the forearm, and after the brachialis muscle is split longitudinally (Figure 4). In distal-third fractures, the lateral column of the distal humerus will be accessed with subperiosteal dissection of the lateral supracondylar crest and reflection of the *brachioradialis* and extensor *carpi radialis longus* muscles and the radial nerve. After indirect reduction under fluoroscopy, a narrow 4.5-mm dynamic compression plate (DCP) will be used and will be introduced in a proximal to distal direction (Figure 5). In fractures of the distal third of the humeral shaft, the plate will be introduced in a distal to proximal direction. Two to three screws will be inserted in each bone fragment. After osteosynthesis, final radiographs will be obtained, and the wound will be sutured and bandaged The patient will be kept immobilised with a sling until ambulatory evaluation.

### Treatment after the intervention

Patients from both of the randomised groups will be included in the same rehabilitation programme. Free-elbow passive and active motion and pendulum exercises for the shoulder will be allowed as soon as the patient can tolerate them. Internal and external shoulder rotations will be introduced 6 weeks after the intervention.

### Outcome assessment

All study participants will be evaluated at 2 and 4 weeks; 2, 6 and 12 months after the intervention. Radiographic evaluations, pain measurements using the visual analogue scale (VAS) [33], and application of the DASH [30,34] (Additional file 3 and Additional file 4), Short Form (SF)-36 and Constant shoulder score questionnaires will be performed by professional orthopaedists and physiotherapists who will not be directly associated with the study. Assessors will be blinded to the treatment assignment. Prior to outcome functional assessments, patients will be instructed not to reveal their treatment and will have their fractured arm covered with an opaque gown, identical in both groups [35].

### Primary outcome

Our primary outcome will be the DASH [30,34] scores 6 months after the procedure (either surgical or nonsurgical) for treatment of humeral shaft fractures. The final score will be calculated using the specified formula:

$$\text{DASH}\;\text{score}=\left(\text{Raw}\;\text{score}-30\right)/1.2.$$

The two optional modules of the DASH questionnaire will not be applied in this study.

### Secondary outcomes

Secondary outcome measures include: (i) the SF-36 questionnaire [36,37], (ii) procedure complications, (iii) pain, measured by the VAS [33], (iv) Constant questionnaire functional score [38]; (v) radiographic characteristics in terms of (a) consolidation of the fracture and (b) displacement and angulation of the fracture fragments.

The SF-36 is a questionnaire containing 36 items. This survey assesses eight health concepts: physical functioning, bodily pain, limitations due to health problems, limitations due to personal or emotional problems, emotional well-being, social functioning, energy/fatigue and general health perceptions. The VAS consists of a 10-cm line anchored by two extremes: ‘no pain’ and ‘pain as bad it could be’. Patients are asked to make a mark on the line, which represents the intensity level of their perceived pain, and the scale is scored by measuring the distance from ‘no pain’ to the patient’s mark.

Consolidation will be considered as a dichotomous variable: fracture healing or no healing. Displacement and angulation of bone fragments will be measured on radiographs.

### Data analysis

Patients, who for any reason demonstrate that their treatment may require additional interventions, will be followed up, and their results will be included in the group into which these patients had initially been randomised, according to the intention-to-treat principle. The Pearson chi-squared test will be used to analyse the results from the two groups in relation to the categorical variables, and Student’s *t*-test will be used to compare the groups in relation to the numerical variables. Student’s *t*-test (parametric) will be used to compare the clinical evolution of each group 2, 4, 8, 24 and 48 weeks after the intervention. For the primary outcome, a significance level of 5% (alpha = 0.05) will be used for all statistical tests, such that tests presenting a *P*-value less than 0.05 will be considered statistically significant. For the secondary outcomes we will consider an alpha value of 0.02.

### Safety

Rates of complications are part of secondary outcome analysis and will be closely monitored. Expected complications in both intervention groups include skin abrasion, skin pressure ulcers, forearm and hand swelling, sensomotor deficit, wound healing problems, hardware displacement and failure, superficial and deep infection, malunion, nonunion, and shoulder and elbow impairment. Complications will be categorized as minor or major according to their severity. Causes of complications will be studied and they will be treated as soon as detected. Complications that may lead to surgical intervention, surgical revision, or clinically important morbidity are classified as severe adverse events. This protocol does not include a data safety monitoring committee.

## Discussion

According to current evidence from a systematic review [28], this study is one of the first randomised controlled trials designed to compare surgical to nonsurgical management of humeral shaft fractures, to evaluate outcomes of quality of life, safety and effectiveness.

Despite the risk of a surgical intervention, the minimally invasive plate osteosynthesis technique seems to be reproducible and applicable in almost all types of humeral shaft fractures. It had the advantage of minimal soft tissue dissection and lower rates of iatrogenic nerve injury when compared to the conventional plate technique. Intramedullary nail osteosynthesis has resulted in higher rates of reoperations and shoulder impairment compared to the compression plate technique [23,25,27]. Functional bracing is a traditional method of treatment but can have some complications, such as, nonunion in proximal-third fractures, residual angulation of more than 10˚ and skin abrasion [16,39].

Fractures too proximal or too distal in the humeral shaft cannot be surgically treated with this minimally invasive technique, which is a limitation of this trial. Another limitation is the fact that this study will take place in only one centre. This not only impacts recruitment, but also may limit the generalisability of the results to other settings. However, the use of broad inclusion criteria is the strength of the trial.

This study aims to provide conclusive, good quality evidence for orthopaedic practice and will contribute to the evidence base of methods used to treat humeral shaft fractures.

## Trial status

This trial started recruiting patients on 6 May 2012.

## Abbreviations

AO: Association for the study of internal fixation; CONSORT: Consolidated standards of reporting trials; DASH: Disability of the arm, shoulder and hand; DCP: Dynamic compression plate; OTA: Orthopaedic trauma association; SF-36: Short form-36; VSA: Visual analogue scale.

## Competing interests

The authors declare that they have no competing interests.

## Authors’ contributions

FTM contributed to trial design, developed the protocol and is the principal investigator of the trial. MJST participated in the conception and design of the trial, drafted the manuscript and acts as coordinating investigator of the trial. MHM participated in development of the trial protocol and coordinates trial monitoring. JBGS and FF participated in the design of the trial and revised the trial manuscript. JCB conceived the trial and acts as coordinating investigator of the trial. All authors contributed to the manuscript and approved the final manuscript.

## Supplementary Material

Additional file 1 Consent for participation in a research (Portuguese). Click here for file

Additional file 2 Consent for participation in a research (English). Click here for file

Additional file 3 DASH questionnaire in Portuguese. Click here for file

Additional file 4 DASH questionnaire in English. Click here for file
