# Supplementary material for: Treatment of the humeral shaft fractures - minimally invasive osteosynthesis with bridge plate versus conservative treatment with functional brace: study protocol for a randomised controlled trial
Source: Trials. 2013 Aug 7;14:246. doi: 10.1186/1745-6215-14-246 (PMC3750574; doi:10.1186/1745-6215-14-246)
Supplement: Additional file 1 — Consent for participation in a research (Portuguese). [file 1745-6215-14-246-S1.pdf]

Consentimento para participação em uma pesquisa

Título da pesquisa: Tratamento das fraturas diafisárias do úmero: osteossíntese com placa em ponte X órtese funcional – Ensaio Clínico Randomizado

Objetivos: o estudo compara dois métodos de tratamento das fraturas diafisárias do úmero, sendo um método conservador e o outro cirúrgico. O objetivo é avaliar qual dos dois métodos de tratamento é mais eficaz.

Procedimentos:

Se eu concordar em participar deste estudo e for selecionado no grupo dos pacientes cirúrgicos acontecerá o seguinte:

1-Serei submetido a um procedimento cirúrgico para colocação de placa e parafusos para a fixação dos fragmentos ósseos.

2-Serão realizadas radiografias do meu braço durante os retornos para acompanhamento da evolução radiológica da fratura.

3-Permanecerei com uma tipóia por sete a dez dias.

4-Farei acompanhamento neste hospital para controle da fratura semanalmente até a sexta semana e após 3,6,12 e 24 meses.

Se eu concordar em participar deste estudo e for selecionado no grupo dos pacientes não cirúrgicos acontecerá o seguinte:

1-Serei tratado com uma imobilização por um período aproximado de

8 semanas

2-Serão realizadas radiografias do meu braço durante os retornos para acompanhamento da evolução radiológica da fratura.

3-Farei acompanhamento neste hospital para controle da fratura semanalmente até a sexta semana e apos 3,6,12 e 24 meses.

Todos estes procedimentos são aceitos pela literatura atual e não ha consenso sobre qual é o melhor método de tratamento.

Não há risco ou desconforto além dos esperados no tratamento das fraturas do úmero. (Exemplos de riscos inerentes ao tratamento das fraturas do úmero: risco anestésico, desconforto pelo uso do imobilizador, dor no local da cirurgia).

Não há benefício direto para o participante. Trata-se de estudo experimental testando a hipótese de que o tratamento cirúrgico é mais eficiente no tratamento das fraturas da diáfise do úmero.

Somente no final do estudo poderemos concluir a presença de algum benefício.

Garantia de acesso: em qualquer etapa do estudo, você terá acesso aos profissionais responsáveis pela pesquisa para esclarecimento de eventuais dúvidas.

O principal investigador é o Dr. Fabio Teruo Matsunaga, que pode ser encontrado no endereço Rua Borges Lagoa 786. Telefone: 11- 5579-7049.

Se você tiver alguma consideração ou dúvida sobre o aspecto ético da pesquisa, entre em contato com o Comitê de Ética em Pesquisa (CEP) – Rua Botucatu, 572 1° andar cj 14, 5571-1062, FAX: 5539-7162. Email: cepunifesp@epm.br.

É garantida a liberdade da retirada de consentimento a qualquer momento e deixar de participar do estudo, sem qualquer prejuízo à continuidade de seu tratamento na Instituição.

Direito de confidencialidade: as informações obtidas serão analisadas em conjunto com outros pacientes, não sendo divulgado a identificação de nenhum paciente.

Direito de ser mantido atualizado sobre os resultados parciais das pesquisas, quando em estudos abertos, ou de resultados que sejam do conhecimento dos pesquisadores.

Despesas e compensações: não há despesas pessoais para o participante em qualquer fase do estudo, incluindo exames e consultas. Também não há compensação financeira relacionada à sua participação.

Se existir qualquer despesa adicional, ela será absorvida pelo orçamento da pesquisa.

Em caso de dano pessoal, diretamente causado pelos procedimentos ou tratamentos propostos neste estudo (nexo causal comprovado), o participante tem direito a tratamento médico na Instituição, bem como às indenizações legalmente estabelecidas.

Compromisso do pesquisador de utilizar os dados e o material coletado somente para esta pesquisa.

Acredito ter sido suficientemente informado a respeito das informações que li ou que foram lidas para mim, descrevendo o estudo "Tratamento das fraturas diafisárias do úmero: osteossíntese com placa em ponte X tratamento não cirúrgico com órtese funcional".

Eu discuti com o Dr. Fabio Teruo Matsunaga sobre a minha decisão em participar nesse estudo. Ficaram claros para mim quais são os propósitos do estudo, os procedimentos a serem realizados, seus desconfortos e riscos, as garantias de confidencialidade e de esclarecimentos permanentes. Ficou claro também que minha participação é isenta de despesas e que tenho garantia do acesso a tratamento hospitalar quando necessário.

Concordo voluntariamente em participar deste estudo e poderei retirar o meu consentimento a qualquer momento, antes ou durante o mesmo, sem penalidades ou prejuízo ou perda de qualquer benefício que eu possa ter adquirido, ou no meu atendimento neste Serviço.

____

Assinatura do paciente / representante legal   Data:____/____ /____

____

Assinatura da testemunha   Data:____/____ /____

Para casos de pacientes analfabetos, semi-analfabetos ou portadores de deficiência auditiva ou visual.

(*Somente para o responsável do projeto*)

Declaro que obtive de forma apropriada e voluntária o Consentimento Livre e Esclarecido deste paciente ou representante legal para a participação neste estudo.

____

Assinatura do responsável pelo estudo   Data: :____/____ /____
