# Supplementary material for: Treatment of the humeral shaft fractures - minimally invasive osteosynthesis with bridge plate versus conservative treatment with functional brace: study protocol for a randomised controlled trial
Source: Trials. 2013 Aug 7;14:246. doi: 10.1186/1745-6215-14-246 (PMC3750574; doi:10.1186/1745-6215-14-246)
Supplement: Additional file 2 — Consent for participation in a research (English). [file 1745-6215-14-246-S2.pdf]

Consent for participation in a research

Title: Treatment of Humeral Shaft Fractures: Minimally Invasive Osteosynthesis with Bridge Plate versus Conservative Treatment with Functional Brace. Randomised Controlled Trial.

Objectives: This study aims to compare two methods of treatment of humeral shaft fractures: one nonsurgical and one surgical method. The objective is to evaluate which of the two methods of treatment is most effective.

Procedures:

If I agree to participate in this study and be selected to surgical treatment group:

1- I will undergo to a surgical procedure, which consist to insert a plate and screws for fixation of bone fragments.

2- Radiographs will be taken from my arm during the returns to monitoring radiological evolution of the fracture.

3- I will stay with a sling for seven to ten days.

4- I will follow this hospital to control the fracture weekly until the sixth weeks and after 3,6,12 and 24 months.

If I agree to participate in this study and be selected to nonsurgical treatment group:

1- I will be treated with a brace for a period of approximately 8 weeks

2- Radiographs will be taken from my arm during the returns to monitoring radiological evolution of the fracture.

3-I will follow this hospital to control the fracture weekly until the sixth weeks and after 3,6,12 and 24 months.

All these procedures are supported by the literature and there is no consensus about which is the best method of treatment.

There will be no risk or discomfort beyond the expected for treatment of humeral fractures. (Examples of risks inherent to the treatment of humeral fractures: anesthaetic risk, discomfort when using the brace, pain at the surgery site, pain at the fracture site).

There is no direct benefit to the participant. This is an experimental study.

Only at the end of the study we can conclude the presence of some benefit.

Guaranteed access: at any stage of the study, you will have access to professionals responsible for research to clarify any doubts.

The principal investigator is Dr. Fabio Matsunaga, which can be found at R. Borges Lagoa 786. Tel: 11 - 5579-7049.

If you have any doubt or consideration about the ethical aspect of the research, please contact the Research Ethics Committee (CEP) - Rua Botucatu 572 1˚And cj 14, 5571-1062, FAX: 5539-7162. Email: cepunifesp@epm.br.

It is guaranteed freedom of withdrawal of consent at any time and stop participating the study with no harm to the continuation of the treatment at the institution.

Right to Confidentiality: the information obtained will be analysed together with other patients, not being disclosed to identify any patient.

Right to be kept updated about the partial results of the research, when in open studies, or results which are known to the researchers.

Expenses and Compensation: no personal expenses for the participant at any stage of the study, including exams and consultations. There is also no financial compensation for their participation.

If there is any additional expense, it will be absorbed by the budget of the research.

In case of personal injury directly caused by the procedures or treatments proposed in this study, the participant is entitled to medical treatment at the institution, as well as the legally established compensation.

Commitment of the researcher to use the data and material collected for this research only.

I believe I have been sufficiently informed about the information that I have read or been read to me, describing the study " *Treatment of Humeral Shaft Fractures: Minimally Invasive Osteosynthesis with Bridge Plate versus Conservative Treatment with Functional Brace*."

I discussed with Dr. Fabio Matsunaga about my decision to participate in this study. It became clear to me what are the purposes of the study, the procedures to be performed, its discomforts and risks, guarantees of confidentiality and clarifications permanent. It also became clear that my participation is free of cost and I have granted access to hospital treatment whenever necessary.

I voluntarily agree to participate in this study and I can withdraw my consent at any time before or during it, without penalty or loss of any benefit or loss that I may have acquired, or in my attendance at this service.

____

Signature of patient / legal representative   Date: ____ / ____ / ____

____

Signature of witness   Date: ____ / ____ / ____

For patient cases illiterate, semi-illiterate or hearing impaired or visually.

(Only for the responsible for the project)

I declare that I obtained appropriately and the voluntary consent of the patient or legal guardian to participate in this study.

____

Signature of the responsible for the study: Date ____ / ____ / ____
