# Supplementary material for: Treatment of the humeral shaft fractures - minimally invasive osteosynthesis with bridge plate versus conservative treatment with functional brace: study protocol for a randomised controlled trial
Source: Trials. 2013 Aug 7;14:246. doi: 10.1186/1745-6215-14-246 (PMC3750574; doi:10.1186/1745-6215-14-246)
Supplement: Additional file 3 — DASH questionnaire in Portuguese. [file 1745-6215-14-246-S3.pdf]

FICHA DE EXTRAÇÃO – PROTOCOLO DE FRATURA DA DIÁFISE DO ÚMERO

Nome: Data:

DASH

	Não houve dificuldade	Houve pouca dificuldade	Houve média dificuldade	Houve muita dificuldade	Não conseguiu fazer
1. Abrir um vidro novo ou com tampa muito apertada	1	2	3	4	5
2. Escrever	1	2	3	4	5
3. Virar uma chave	1	2	3	4	5
4. Preparar uma refeição	1	2	3	4	5
5. Abrir uma porta pesada	1	2	3	4	5
6. Colocar algo em uma prateleira acima de sua cabeça	1	2	3	4	5
7. Fazer tarefas domésticas pesadas (lavar paredes, lavar o chão)	1	2	3	4	5
8. Fazer trabalho de jardinagem	1	2	3	4	5
9. Arrumar a cama	1	2	3	4	5
10. Carregar uma sacola ou uma maleta	1	2	3	4	5
11. Carregar um objeto pesado (mais de 5kg)	1	2	3	4	5
12. Trocar uma lâmpada acima da cabeça	1	2	3	4	5
13. Lavar ou secar o cabelo	1	2	3	4	5
14. Lavar suas costas	1	2	3	4	5
15. Vestir uma blusa fechada	1	2	3	4	5
16. Usar uma faca para cortar alimentos	1	2	3	4	5
17. Atividades recreativas que exigem pouco esforço (cartas, tricotar)	1	2	3	4	5
18. Atividades recreativas que exigem força/impacto (vôlei, martelar)	1	2	3	4	5
19. Atividades recreativas que movem braço livre/ (pescar, peteca)	1	2	3	4	5
20. Transportar-se de um lugar a outro (ir de um lugar a outro)	1	2	3	4	5
21. Atividades sexuais	1	2	3	4	5

	Não afetou	Afetou pouco	Afetou medianamente	Afetou muito	Afetou extremamente
22. Na semana passada, em que ponto o seu problema com braço, ombro ou mão afetou suas atividades normais com família, amigos, vizinhos ou colegas?	1	2	3	4	5

	Não limitou	Limitou pouco	Limitou medianamente	Limitou muito	Limitou extremamente
23. Durante a semana passada, o seu trabalho ou atividades diárias normais foram limitadas devido ao seu problema com braço, ombro ou mão?	1	2	3	4	5

Meça a gravidade dos seguintes sintomas na semana passada:	Nenhuma	Pouca	Mediana	Muito	Extrema
24. Dor no braço, ombro ou mão	1	2	3	4	5
25. Dor no braço, ombro ou mão quando você fazia atividades específicas	1	2	3	4	5
26. Desconforto na pele (alfinetadas) no braço, ombro ou mão	1	2	3	4	5
27. Fraqueza no braço, ombro ou mão	1	2	3	4	5
28. Dificuldade em mover braço ombro ou mão	1	2	3	4	5

	Não houve dificuldade	Pouca dificuldade	Média dificuldade	Muita dificuldade	Tão difícil que você não pôde dormir
29. Durante a semana passada, qual a dificuldade que você teve para dormir por causa da dor no seu braço, ombro ou mão?	1	2	3	4	5

	Discordo totalmente	Discordo	Não concordo ne discordo	Concordo	Concordo totalmente
30. Eu me sinto menos capaz, menos confiante e menos útil por causa do meu problema com braço, ombro ou mão	1	2	3	4	5
