# Supplementary material for: Treatment of the humeral shaft fractures - minimally invasive osteosynthesis with bridge plate versus conservative treatment with functional brace: study protocol for a randomised controlled trial
Source: Trials. 2013 Aug 7;14:246. doi: 10.1186/1745-6215-14-246 (PMC3750574; doi:10.1186/1745-6215-14-246)
Supplement: Additional file 4 — DASH questionnaire in English. [file 1745-6215-14-246-S4.pdf]

DASH

Rate your ability of doing the following activities last week by circling the corresponding number:

	Not difficult	A little difficult	Average difficulty	Very difficult	Unable
1. Open a tight jar	1	2	3	4	5
2. Write	1	2	3	4	5
3. Turn a key	1	2	3	4	5
4. Cook a meal	1	2	3	4	5
5. Push open a heavy door	1	2	3	4	5
6. Place an object on a shelf above your head	1	2	3	4	5
7. Do heavy housework (wash walls, wash floor)	1	2	3	4	5
8. Gardening	1	2	3	4	5
9. Make the bed	1	2	3	4	5
10. Carry a heavy bag or a suitcase	1	2	3	4	5
11. Carry a heavy object (more than 5 kg)	1	2	3	4	5
12. Change a light bulb above your head	1	2	3	4	5
13. Wash or dry your hair	1	2	3	4	5
14. Wash your back	1	2	3	4	5
15. Put on a sweater	1	2	3	4	5
16. Use a knife to cut food	1	2	3	4	5
17. Recreational activities (play cards, knit)	1	2	3	4	5
18. Recreational activities, which cause impact in your arms, shoulders and hands (play volleyball, hammering)	1	2	3	4	5
19. Recreational activities in which you move your arm freely (fishing, badminton)	1	2	3	4	5
20. Handle transportation needs	1	2	3	4	5
21. Sexual activities	1	2	3	4	5

	Nothing	A little	Average	A lot	Extremely
22. Last week, how much has your problem affected your regular activities with family, friends, neighbors or groups?	1	2	3	4	5

	No	A little	Average	A lot	Unable
23. During last week, was your work or regular activities limited because of your problem?	1	2	3	4	5

Rate the severity of the following symptoms last week:	None	A little	Average	A lot	Extreme
24. Pain in the arm, shoulder or hand	1	2	3	4	5
25. Pain in the arm, shoulder or hand while doing specific activities	1	2	3	4	5
26. Pins and needles in your arm, shoulder or hand	1	2	3	4	5
27. Weakness in the arm, shoulder or hand	1	2	3	4	5
28. Difficulty in moving the arm, shoulder or hand	1	2	3	4	5

	Not difficult	Not very difficult	Average difficulty	Very difficult	So difficult that I couldn't sleep
29. During last week, how difficult was it for you to sleep because of the pain in your arm, shoulder or hand?	1	2	3	4	5

	Totally disagree	Disagree	Neither agree or disagree	Agree	Totally agree
30. I feel less capable, confident and useful because of my problem	1	2	3	4	5
